# Late Pleistocene polar bear genomes reveal the timing of allele fixation in key genes associated with Arctic adaptation

**DOI:** 10.1186/s12864-024-10617-3

**Published:** 2024-09-16

**Authors:** Yulin Sun, Eline D. Lorenzen, Michael V. Westbury

**Affiliations:** 1 Globe Institute, University of Copenhagen, Copenhagen, Denmark; 2 School of The Environment, The University of Queensland, Brisbane, QLD Australia

**Keywords:** Adaptation, Ancient DNA, Arctic, Genomics, Polar bear

## Abstract

The polar bear (*Ursus maritimus*) occupies a relatively narrow ecological niche, with many traits adapted for cold temperatures, movement across snow, ice and open water, and for consuming highly lipid-dense prey species. The divergence of polar bears from brown bears (*Ursus arctos*) and their adaptation to their Arctic lifestyle is a well-known example of rapid evolution. Previous research investigating whole genomes uncovered twelve key genes that are highly differentiated between polar and brown bears, show signatures of selection in the polar bear lineage, and are associated with polar bear adaptation to the Arctic environment. Further research suggested fixed derived alleles in these genes arose from selection on both standing variation and de novo mutations in the evolution of polar bears. Here, we reevaluate these findings based on a larger and geographically more representative dataset of 119 polar bears and 135 brown bears, and assess the timing of derived allele fixation in polar bears by incorporating the genomes of two Late Pleistocene individuals (aged 130–100,000 years old and 100–70,000 years old). In contrast with previous results, we found no evidence of derived alleles fixed in present-day polar bears within the key genes arising from de novo mutation. Most derived alleles fixed in present-day polar bears were also fixed in the Late Pleistocene polar bears, suggesting selection occurred prior to 70,000 years ago. However, some derived alleles fixed in present-day polar bears were not fixed in the two Late Pleistocene polar bears, including at sites within *APOB*, *LYST*, and *TTN*. These three genes are associated with cardiovascular function, metabolism, and pigmentation, suggesting selection may have acted on different loci at different times.

**Supplementary Information:**

The online version contains supplementary material available at 10.1186/s12864-024-10617-3.

## Introduction

The polar bear (*Ursus maritimus*) is uniquely adapted to the extreme conditions of life in the High Arctic and spends most of its life out on sea ice. In cold Arctic climates, energy is in high demand. As a result, the polar bear feeds on a lipid-rich diet throughout its life [1]. Polar bears are most closely related to the brown bear (*Ursus arctos*), a widely distributed omnivore found in a variety of habitats across the Holarctic [2]. The two species differ fundamentally in their ecology, behaviour, and morphology, reflecting adaptations to different ecological niches. Polar bears diverged from brown bears relatively recently – within the past ~ 1,000,000 years [3–5].

A previous study reported twelve key genes showing a signal of strong positive selection in the polar bear lineage [3]. These genes may have played significant roles in the ability of polar bears to rapidly adapt to their new Arctic environment. They included *APOB*, *LYST*, and *TTN*, which are related to cardiovascular functions (*APOB, TTN*), metabolism (*APOB, LYST*), and pigmentation (*APOB, LYST*).

**Fig. 1:**
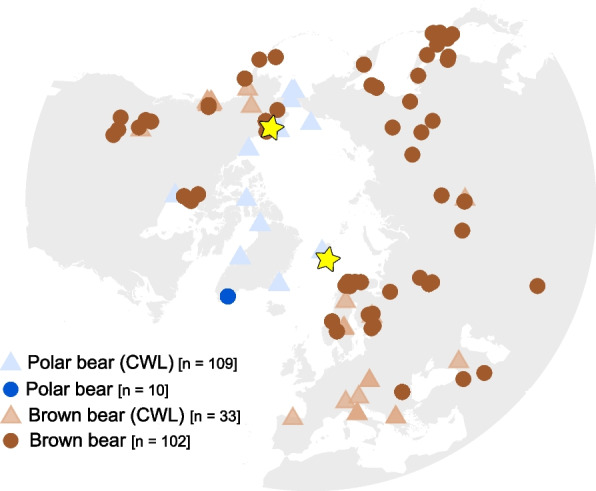
Geographic localities of the polar and brown bears included in this study. CWL shows the bears that were used in the study by Castruita, Westbury, and Lorenzen [6]. Stars indicate the two Late Pleistocene polar bears

Further research utilising the 109 polar and 33 brown bear genomes available at the time (Fig. 1) investigated whether derived alleles fixed in eleven of those same genes identified as under selection in the polar bear lineage were due to selection on standing variation, or on de novo mutations. Evidence was found of both, suggesting variation present in the ancestral polar/brown bear gene pool and de novo mutations played a role in the evolution of polar bears [6].

Here, we build on this work by incorporating additional, recently published polar bear (*n* = 10) and brown bear (*n* = 102) genomes from previously unstudied populations [7–9] (Fig. 1). The expanded geographic coverage of our data allowed us to more generally characterise whether the fixed derived alleles in the previously identified key genes [3] originate from standing variation or de novo mutation. By analysing a larger dataset [7–9], we minimise the possibility of missing data and/or population structure having influenced previous inferences. Furthermore, we incorporate genomic data from two Late Pleistocene polar bears aged 130–100,000 years old (‘Poolepynten’, Svalbard) and 100–70,000 years old (‘Bruno’, Alaska) [2, 10], to investigate the timing of fixation. Establishing a reliable time frame for when derived alleles in the previously identified key genes [3] become fixed can improve our understanding of what evolutionary processes drove speciation, and the rates in which novel adaptations to extreme environments can arise.

## Results

### Ancient DNA damage in the Late Pleistocene polar bears

Investigations into whether the ancient DNA (aDNA) found in Poolepynten and Bruno were authentic showed typical DNA degradation patterns. We observed high levels of C-T substitution towards the ends of the reads and G-A on the reverse complement in both Late Pleistocene samples (Supplementary Fig. 1). Bruno displayed less damage, with ~ 5% of the sites at the read ends experiencing aDNA damage. Poolepynten had more damage, with ~ 20% of the sites at the read ends showing aDNA damage patterns.

### Genomic differentiation

As gene flow is known to occur between polar and brown bears [4, 11], we investigated whether the genes and their surrounding regions were still highly differentiated between polar and brown bears, despite the increase in sample size. To do this we performed independent principal component analysis for each gene, including their 50 kb flanking regions. In all eleven principal component analyses we observed clear differentiation between polar and brown bear individuals, suggesting no interspecific admixture at these loci (Supplementary Figs S2-S12). We note that a lack of differentiation between polar and brown bears in the loci could not only be caused by gene flow, but also other factors such as incomplete lineage sorting. However, the main purpose of this analysis was to see if these regions were still differentiated; if not, they could not be considered responsible for the phenotypic differences observed between polar and brown bears.

### Fixed derived alleles in polar bears

**Fig. 2:**
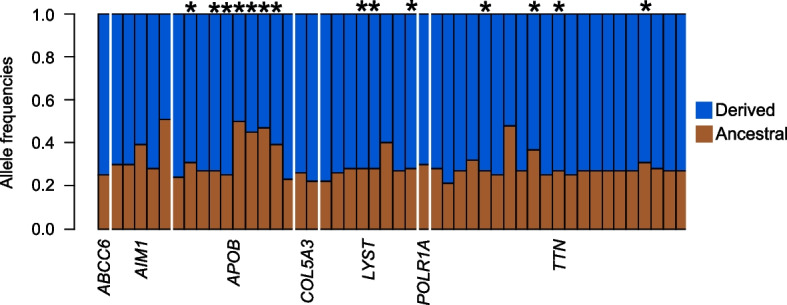
Allele frequencies of derived and ancestral alleles in brown bears at 48 sites where all polar bears are fixed for the derived allele. Asterisks show sites where at least one of the two Late Pleistocene polar bears are not fixed for the derived allele. Four genes (*CUL7, FCGBP, LAMC3, XIRP1*) are not shown, as no sites were fixed for the derived allele in present-day polar bears. The analysis was based on 119 present-day polar bears and 135 brown bears, and the two Late Pleistocene polar bear individuals

To understand whether selection may have occurred on standing variation or de novo mutations in the polar bear lineage, we investigated the presence of fixed derived alleles in the polar bear lineage. We consider a derived allele as de novo in the polar bear lineage if it is not found in either the brown bear, or the outgroups. When comparing genotype calls between all present-day polar and brown bears, we found no sites fixed for the polar bear reference genome allele in polar bear, and fixed for the alternative allele in the brown bear, giant panda (*Ailuropoda melanoleuca*), and spectacled bear (*Tremarctos ornatus*) (Fig. 2, Supplementary Table S1). Thus, we found no evidence for any of the fixed derived alleles in the eleven focus genes to have arisen by de novo mutations in the polar bear lineage. Four genes (*CUL7, FCGBP, LAMC3, XIRP1*) contained no sites fixed for the derived allele in the present-day polar bears. A lack of fixed derived alleles suggests no specific allele within said gene was a necessity in the evolution of polar bears. We therefore did not consider these genes in future interpretations. In the remaining seven genes, we found 48 sites that were fixed for the derived allele in all present-day polar bears.

### Timing of allele fixation in polar bears

To determine the timing of allelic fixation, we investigated whether the two Late Pleistocene polar bears were also fixed for the derived allele, as seen in the present-day polar bears. Seven genes remained after filtering out the gene that was previously shown to not be highly differentiated between species (*EDH3*) [6] and the four genes that did not contain any fixed derived alleles (*CUL7, FCGBP, LAMC3, and XIRP1*). These seven genes had a total of 34 sites where the Late Pleistocene individuals were also fixed for the derived allele (Fig. 2, Table 1, Supplementary Table S1). Only *APOB, LYST* and *TTN* contained sites (a total of 14) where at least one of the Late Pleistocene individuals also contained the ancestral allele (Table 1, Supplementary Table S1).

**Table 1:** Timing of fixation of derived alleles in seven genes previously identified to show the strongest signals of selection in the polar bear lineage [3]. Genes with no fixed derived alleles in polar bears after filtering are not shown. Associated phenotypes are from the MGI database [12]

Gene	Associated Phenotype	Coding Length (bp)	Number of alleles fixed before the age of Late Pleistocene individuals	Number of alleles fixed after the age of Late Pleistocene individuals
*ABCC6*	Cardiovascular	4551	1	0
*AIM1*	Pigmentation	5484	5	0
*APOB*	Cardiovascular, metabolism, pigmentation	13,305	3	7
*COL5A3*	Adipose tissue, metabolism	5256	2	0
*LYST*	Metabolism, pigmentation	11,403	5	3
*POLR1A*	Cardiovascular	5172	1	0
*TTN*	Cardiovascular	102,861	17	4

### Assessing heterozygous base call reliability

As ancient DNA damage can lead to an increase in C-T and A-G transitions, we considered whether these could bias our results by increasing the number of heterozygous sites in the Late Pleistocene polar bears. Therefore, we set the threshold of minor allele frequency of 30% to determine whether a heterozygous base call in the Late Pleistocene individuals was a false positive. We found 14 heterozygous sites in the Late Pleistocene individuals (Supplementary Table S2), two of which may be false positives in the *TTN* gene (minor allele frequencies of 25% and 29%). However, through a manual visualisation of the mapped reads we found the minor alleles always occurred within the read as opposed to the end. As aDNA damage occurs mostly towards the ends of the reads, and they were close to the 30% cutoff, we designated these as true heterozygous sites.

## Discussion

By including the genomes of two Late Pleistocene polar bears in an analysis of 119 present-day polar bears and 135 present-day brown bears (Fig. 1), we infer when derived alleles in genes previously proposed to have been key in polar bear evolution [3], become fixed in the polar bear lineage.

We identified 34 sites fixed for the derived allele in all polar bears – present-day and Late Pleistocene – suggesting fixation of these derived alleles occurred prior to the ages of the Late Pleistocene polar bears (> 130,000 years ago). This is congruent with morphometric measurements of Poolepynten, which is a well-preserved mandible. In comparison with present-day and other fossil polar bears, as well as brown bears, Poolepynten falls within the range of present-day polar bears [13]. Stable isotopes also revealed it to be subsisting on a marine diet. Therefore, we can assume this individual already possessed key polar bear traits and was adapted to the Arctic environment.

However, we also identified 14 sites with derived alleles that were fixed in all present-day polar bears but not in both Late Pleistocene bears, suggesting their fixation occurred after the age of the Late Pleistocene polar bears (< 70,000 years ago). These derived alleles were found in only three investigated genes: *APOB, LYST*, and *TTN*. Although it is difficult to identify the determinant allele for a phenotype, this result suggests these alleles may not have been important initially for polar bear adaptation to an Arctic existence. However, as these three genes have broad overlapping associations with the cardiovascular system, metabolism, and pigmentation (Table 1), as the other genes investigated (*ABCC6, AIM1, COL5A3, POLR1A*), we suggest they may have played a later but also vital role in refining polar bears’ Arctic adaptation.

The gene showing the highest number of derived alleles (7/10), which our analyses suggest became fixed more recently, was *APOB* (Fig. 2). *APOB* encodes apolipoprotein B (apoB), which is associated with the cardiovascular system [14]. It has been suggested that selection on the *APOB* gene may have played a role in the novel adaptation of polar bears to a lipid-rich diet, and increased the efficacy of cholesterol clearance from the blood [3, 6]. The feeding ecology of Poolepynten was shown to fall within the range of present-day polar bears, who prey mainly on ringed seals and bearded seals [13]. Therefore, we can assume that the ability to process a lipid-rich diet was required more than 70,000 years ago, suggesting selection cannot have driven this phenotype within the last 70,000 years. This could suggest that the variants we discuss here may not have been essential in the early adaptation of polar bears, but may have been driven by increased selective pressures during the later stages of the last glacial period. Other genes previously shown to have strong signals of selection in the polar bear lineage [3], such as *ABCC6, POLR1A* and *COL5A3*, also have functions related to the cardiovascular system and metabolism [6, 12]. As these genes only have derived alleles fixed in the Late Pleistocene and present-day polar bears, they may have played a key role in driving the early adaptation of polar bears to a lipid-rich diet.

Similar to *APOB, TTN* is associated with the cardiovascular system. *TTN* encodes Titin, an abundant protein of striated muscle, which includes cardiac muscle tissue; mutations in *TTN* are linked with human cardiac physiology [15]. The genes *AIM1* and *LYST* are both associated with pigmentation [16, 17]. Pigmentation is not preserved in the fossil record, which consists only of skeletal remains, and thus we have no pre-historic evidence of a white phenotype. In *LYST*, the majority of fixed derived alleles in present-day polar bears (five alleles) are also fixed in the Late Pleistocene polar bears. The three alleles fixed in present-day polar bears, but not in the Late Pleistocene polar bears, may have been driven to fixation in the former by selection in the last ~ 70,000 years, or by linkage disequilibrium.

In contrast with previous findings [6], we did not observe any indication of de novo mutation in the eleven genes investigated. All derived alleles fixed in present-day polar bears were present in brown bears, suggesting their presence in the ancestral brown/polar bear gene pool. The increase from 33 to 135 brown bear individuals in this study relative to previous work (Fig. 1) decreased the chances of allelic drop out. As polar bears rapidly adapted to their novel Arctic environment, the lack of de novo mutations in the polar bear lineage is perhaps not surprising. While standing variation and de novo mutation both provide the raw material for evolution, standing variation is already present in the gene pool for selection to act upon, allowing for immediate use in adaptation. De novo mutations arise randomly, segregate at an initially low frequency, and therefore require more time to reach fixation under the same selective pressure [18]. Thus, standing variation was key to the ability of polar bears to survive the Arctic environment – no matter when selection occurred. De novo mutations that would convey a selective advantage may not have been rapid enough during their transition to the Arctic. This result supports that maintaining high levels of standing variation is key to the long-term survival of a species, and may aid in their adaptation to rapidly changing environments.

Our study provides novel evidence of the timing and modes of selection in the polar bear lineage, but is not without caveats. We mapped all raw reads to the polar bear reference genome. Therefore, there may be a bias towards the reference allele [19, 20] and decreased mapping efficiency for the more distant outgroup individuals [21], which may cause some relevant sites to not be considered. However, as polar and brown bears are relatively closely related [3–5], and as we only considered individuals if the site of interest had a minimum read depth of four, we do not think reference bias would have played a large role in our inferences. We focused on derived allele fixation within coding regions of the genome. However, novel mutations in non-coding regions, e.g. in regulatory elements, may have also played a role and are an interesting avenue for future research. Palaeogenomic data are only available from two Late Pleistocene polar bear individuals. Consequently, inferences regarding the timing of allele fixation must be interpreted with caution, especially when it comes to the generalisability of the alleles fixed in the Late Pleistocene individuals and present-day polar bears. Specifically, the fixation of a given allele in only two individuals cannot be confidently extrapolated to the wider polar bear population that existed during the Late Pleistocene, although our two individuals are geographically disparate and therefore may be representative of polar bear ancestry at the time. Despite this, we are more confident that if an allele is not fixed in the two Late Pleistocene polar bears, then it is highly likely to have only become fixed after the age of the youngest specimen (70,000 years ago), as we observe for alleles in *APOB, LYST, TTN*. As additional ancient data from a wider temporal and spatial array of polar bears may become available in future, it may be possible to further pinpoint the timing of allelic fixation and these crucial adaptations, which have enabled polar bears to inhabit one of the coldest environments on Earth.

## Methods

### Late Pleistocene polar bear individuals

Available data from two Pleistocene polar bears were included in the study. Genomic data of Bruno (110–70,000 years old) was previously generated from the skull of a juvenile polar bear sample that was found in 2009 on the coast of the Beaufort Sea, near Point McLeod in Arctic Alaska [2]. Genomic data from Poolepynten (130–110,000 years old) was previously extracted from a left mandible, which was found in Svalbard [10, 13]. Age determination with infrared-stimulated luminescence suggested that it is probably the oldest polar bear fossil discovered [10, 22].

### Present-day individuals

Following the previous study by Castruita, Westbury, and Lorenzen [6], our analysis included the data set from Liu et al. of 89 genomes [3] (79 polar bear, 10 brown bear) and the 30 polar bear and 23 brown bear genomes published elsewhere [11, 23–26]. We obtained the mapped files from the Castruita, Westbury, and Lorenzen publication which utilised raw reads from NCBI (Bioproject IDs: PRJNA169236, PRJNA196978, PRJNA210951, PRJNA271471, PRJNA395974, and PRJEB27491).

New to the present study, we incorporated available genomic data from populations of polar bears in Southeast Greenland (*n* = 10) [7], and brown bears from Hokkaido, Japan (*n* = 6) [9] and across their Holarctic distribution (*n* = 96) [8]. We downloaded the SRA files from NCBI from the Bioproject IDs: PRJNA669153, PRJDB11280, and PRJNA913591. Information on the newly incorporated individuals can be found in Supplementary Table S3.

### Raw data processing

For the 142 individuals from Castruita, Westbury, and Lorenzen [6], raw sequencing reads were previously processed with the PALEOMIX [27] pipeline. Internally, adapter sequences, stretches of Ns, and low-quality bases were trimmed and filtered with AdapterRemovalv2 [28] using default parameters. BWA v0.7.15 [29] aln was used to map the cleaned reads to the pseudo-chromosomal polar bear genome (Genbank accession: GCA_000687225.1) from Liu et al. [3], with default parameters. We chose the pseudo-chromosome assembly as the reference genome to keep our analyses consistent with the previous studies [3, 6]. Reads with mapping quality of less than 30 were filtered using SAMtools v1.6 [30]. Duplicates were removed with picard v2.6.0 [31]. Possible paralogs were filtered using SAMtools. Finally, local realignment around indels was performed using GATK (v 3.3) [32].

For the 112 newly incorporated individuals, we trimmed adapter sequences and polyG sequences (-g) and merged overlapping read pairs (-m) with Fastp v0.23.2 [33]. To the exclusion of the -g and -m parameters, we otherwise used default parameters. We mapped the cleaned reads to the same pseudochromosome polar bear genome with BWA v0.7.15 [29] aln with the seed disabled (-l 690). We used SAMtools v1.6 [30] to filter the reads with mapping quality of less than 30 and remove duplicates. We assessed aDNA damage in the two Late Pleistocene polar bear individuals and adjusted base quality scores around damage using Mapdamage2 (–rescale) [34, 35]. To determine the ancestral allele, we included single individual representatives of the spectacled bear [36] and the giant panda [37] (Bioprojects PRJNA472085 and PRJNA38683). We mapped the reads to the same pseudochromosome polar bear genome following the same approach as the 112 newly incorporated present-day individuals.

### Genomic differentiation

To investigate whether there was still clear genomic differentiation between polar bears and brown bears at the eleven genes of interest, we performed independent principal component analyses (PCAs) for each gene including the 50 kb regions upstream and downstream of the gene. We used a genotype likelihood approach to construct the PCAs: input genotype likelihood files were constructed using ANGSD v0.929 [38], with the SAMtools genotype likelihood algorithm (− GL 1), and specifying the following parameters: remove reads that have multiple mapping best hits (− uniqueonly), remove reads with a flag above 255/secondary hits (− remove_bads), include only read pairs with both mates mapping correctly (− only_proper_pairs), adjust mapQ for reads with excessive mismatches (− C 50), adjust quality scores around indels (− baq 1), a minimum mapping quality of 20 (− minMapQ 20), a minimum base quality of 20 (− minQ 20), determine the major allele based on the genotype likelihoods (-doMajorMinor 1), calculate allele frequencies assuming a fixed major allele and an unknown minor allele (-doMaf 2), generate beagle output file (-doGlf 2), discard sites where there is no data in at least 95% of the individuals (− minInd), skip tri-allelic sites (− skipTriallelic), and remove SNP sites with a *p*-value larger than 1e − 6 (− SNP_pval 1e-6). The ANGSD output beagle file was run through PCAngsd v0.95 [39] to generate a covariance matrix.

### Genotype calling

We investigated eleven of the genes previously inferred using population genomics and demographic modelling to have the strongest signals of positive selection in the polar bear [3]. These included *ABCC6, AIM1, APOB, COL5A3, CUL7, FCGBP, LAMC3, LYST, POLR1A, TTN*, and *XIRP1*. We excluded *EDH3* due to potential for admixture between polar and brown bears in the genomic region containing the gene [6].

We called genotypes using ANGSD v0.921 [38] following the approach of [6]. To call genotypes we used the SAMtools genotype likelihood algorithm (-GL 1) and the following parameters; remove reads that have multiple mapping best hits (-unique_only 1), remove reads with a flag above 255/secondary hits (-remove_bads 1), adjust quality scores around indels (-baq 1), a minimum mapping quality of 20 (-minMapQ 20), a minimum base quality of 20 (-minQ 20), write major and minor alleles and the genotype directly (-doGeno 5), estimate the posterior genotype probability based on the allele frequency as a prior (-doPost 1), use the reference allele as the major allele (-doMajorMinor 4), and calculate allele frequencies assuming a fixed major allele and an unknown minor allele (-doMaf 2). In order to decrease biases that could arise when calling heterozygous alleles from the low-coverage genomes, we only called genotypes from individuals that had at least 4 × coverage at the site of interest (-geno_minDepth 4). We only included biallelic sites where each allele led to a different amino acid (non-synonymous differences).

To determine which allele was the ancestral allele, we used the outgroup spectacled bear and giant panda sequences. If the allele fixed in all polar bears was found in either of these individuals, we removed that site from further consideration.

We further investigated for false positive heterozygous sites that may have arisen due to aDNA damage in the Late Pleistocene individuals. We investigated the read count for each of the four bases at each site of interest, focusing on heterozygous sites which might be caused by aDNA damage (C-T and G-A). Read counts were generated in ANGSD using the -dumpcount parameter. We calculated the proportion of the minor base of each heterozygous site and only if the ratio is more than 30%, would we assume that this site is heterozygous and not a false positive.

## Supplementary Information

Supplementary Material 1.

Supplementary Material 2.

## Data Availability

All polar and brown bear short read data can be found under the following NCBI Bioproject IDs: PRJNA169236, PRJNA196978, PRJNA210951, PRJNA271471, PRJNA395974, PRJEB27491, PRJNA669153, PRJDB11280, and PRJNA913591. The polar bear genome used as the mapping reference can be found under the Genbank accession: GCA_000687225.1. The pseudo-chromosome version of the above polar bear genome produced by Liu et al. 2013, can be found on the University of Copenhagen’s Electronic Research Data Archive (ERDA) under the following link: https://sid.erda.dk/share_redirect/amLYDcI3uJ The spectacled bear and giant panda short read data found under the following NCBI Bioproject IDs PRJNA472085 and PRJNA38683.
