# Supplementary material for: Late Pleistocene polar bear genomes reveal the timing of allele fixation in key genes associated with Arctic adaptation
Source: BMC Genomics. 2024 Sep 16;25:826. doi: 10.1186/s12864-024-10617-3 (PMC11403954; doi:10.1186/s12864-024-10617-3)
Supplement: Supplementary file 1 — Supplementary Material 1. [file 12864_2024_10617_MOESM1_ESM.pdf]

Supplementary Information

Supplementary tables

Table S1. Gene location, nucleotide information, allele counts, and amino acid information for the seven genes of interest. ‘R’ indicates the reference allele count. ‘A’ indicates the ancestral allele count. Amino acid information taken from Castruita, Westbury, and Lorenzen 2020. Four genes (*CUL7, FCGBP, LAMC3, XIRP1*) are not shown due to a lack of fixed derived alleles in the polar bear.

Table S2. Heterozygous sites (marked by yellow), base counts, and the proportion of the minor allele at the highlighted heterozygous site. The base count is only shown for the heterozygous individual.

Table S3: Geography, genome-wide mean coverage, and reference for the newly included polar and brown bear genomes, including the two ancient polar bears.

Supplementary figures

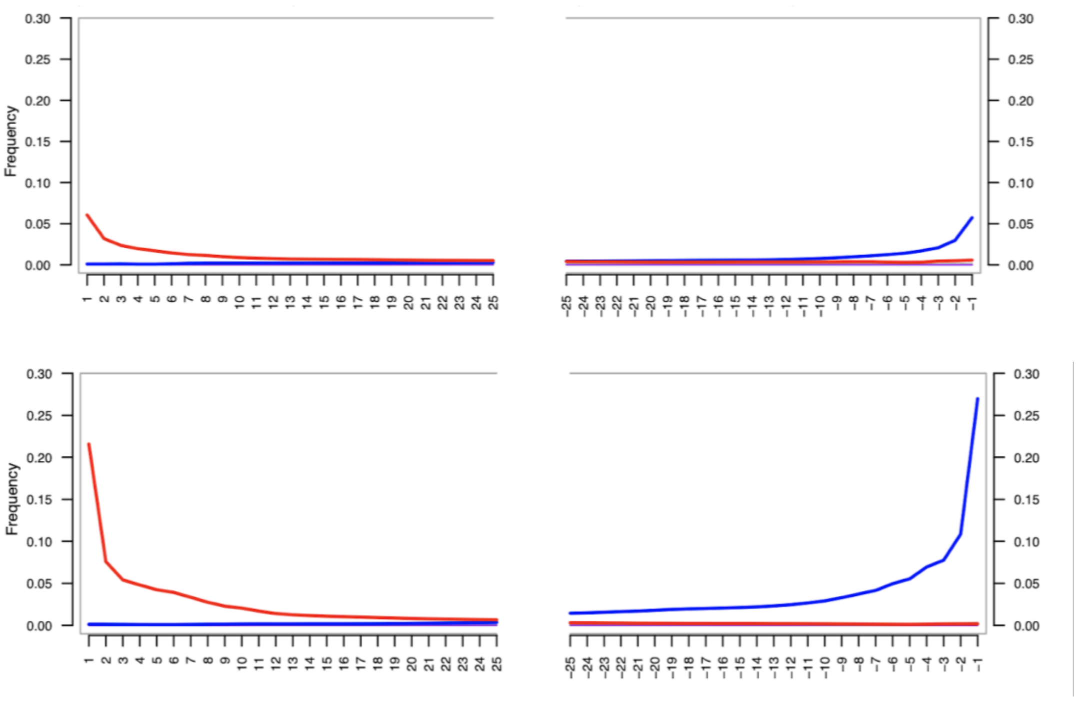

Figure S1. Mapdamage results of the two Late Pleistocene polar bear individuals. Upper plot shows the damage patterns for Bruno, lower plot shows the damage patterns for Poolepynten. Red indicates C to T transitions, blue indicates G to A transitions. Y -axis denotes the proportions of sites containing a nucleotide change from the reference sequence. X-axis represents position from 5' (left) and 3' (right) read end.

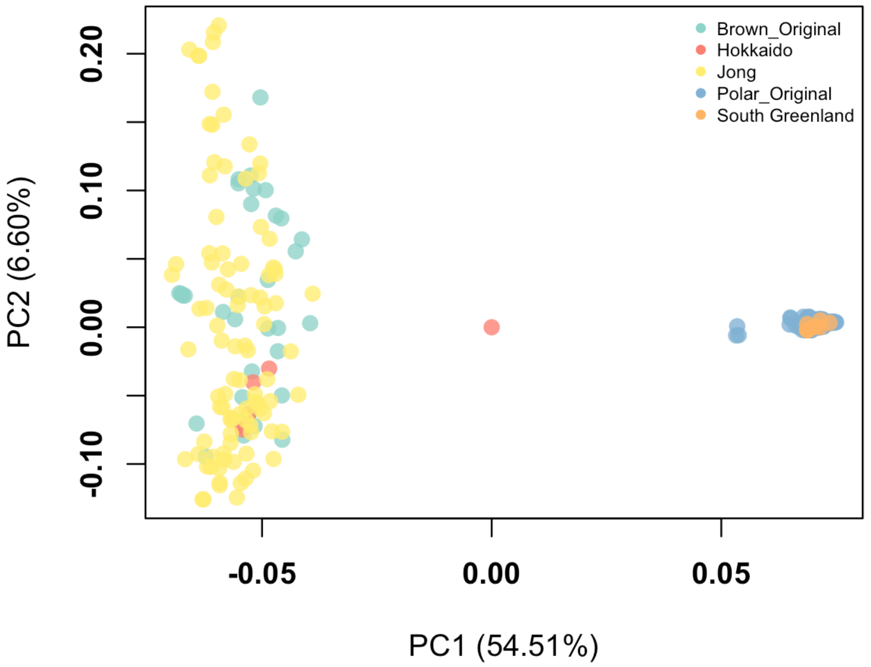

Figure S2. Principal component analysis of gene *AIM1* and the 50kb flanking regions using all present-day individuals (119 polar bears, 135 brown bears) included in this study. ‘Polar_Original’ and ‘Brown_Original’ refer to the samples from Castruita, Westbury, and Lorenzen 2020. ‘South Greenland’, ‘Hokkaido’, represent new polar bear datasets from Laidre et al 2023 and Endo et al 2021, respectively. ‘Jong’ shows the Holarctic brown bear dataset from de Jong et al 2023.

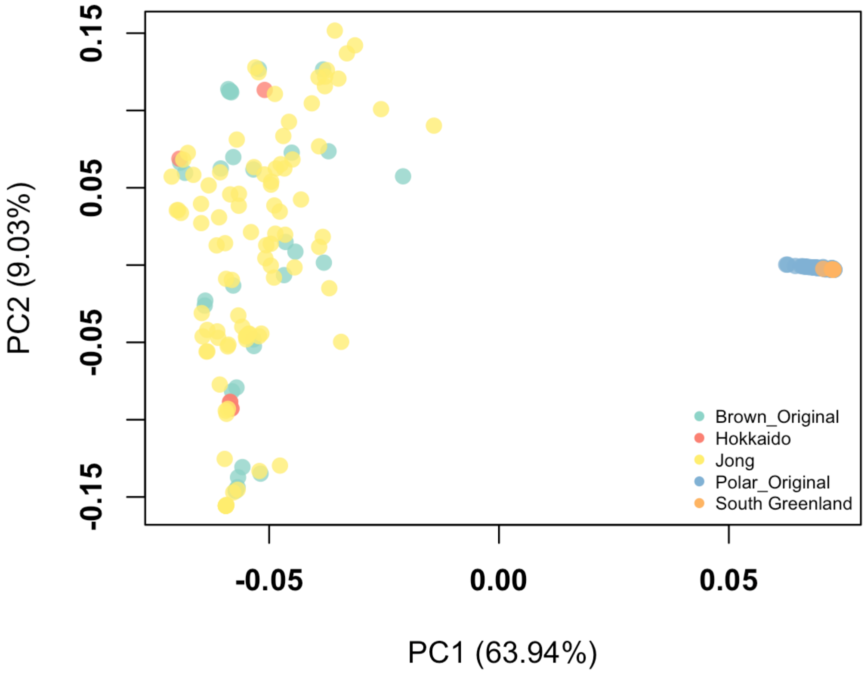

Figure S3. Principal component analysis of gene *ABCC6* and the 50kb flanking regions using all present-day individuals (119 polar bears, 135 brown bears) included in this study. 'Polar_Original' and 'Brown_Original' refer to the samples from Castruita, Westbury, and Lorenzen 2020. 'South Greenland', 'Hokkaido', represent new polar bear datasets from Laidre et al 2023 and Endo et al 2021, respectively. 'Jong' shows the Holarctic brown bear dataset from de Jong et al 2023.

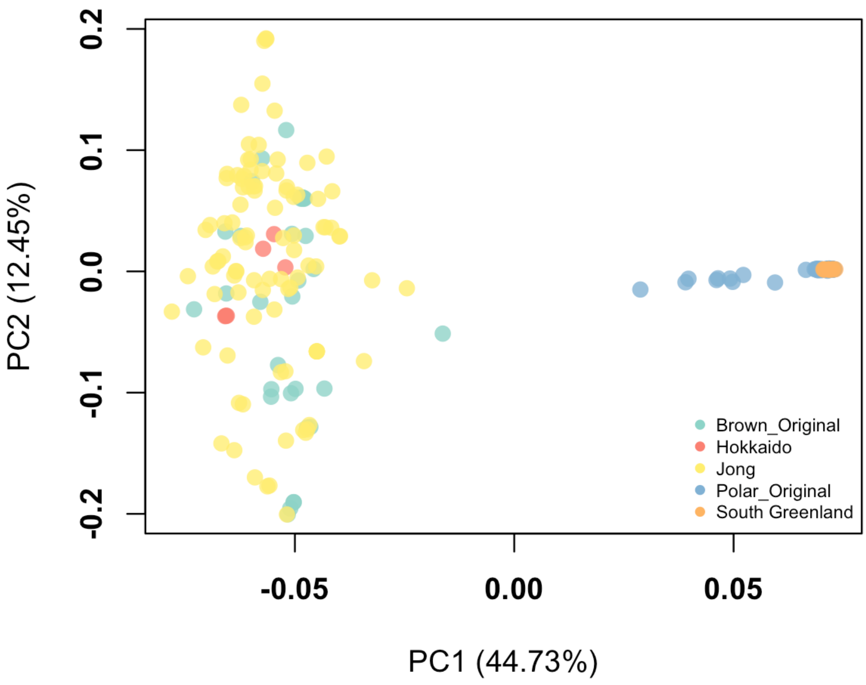

Figure S4. Phe principal component analysis of gene *APOB* and the 50kb flanking regions using all present-day individuals (119 polar bears, 135 brown bears) included in this study. ‘Polar_Original’ and ‘Brown_Original’ refer to the samples from Castruita, Westbury, and Lorenzen 2020. ‘South Greenland’, ‘Hokkaido’, represent new polar bear datasets from Laidre et al 2023 and Endo et al 2021, respectively. ‘Jong’ shows the Holarctic brown bear dataset from de Jong et al 2023.

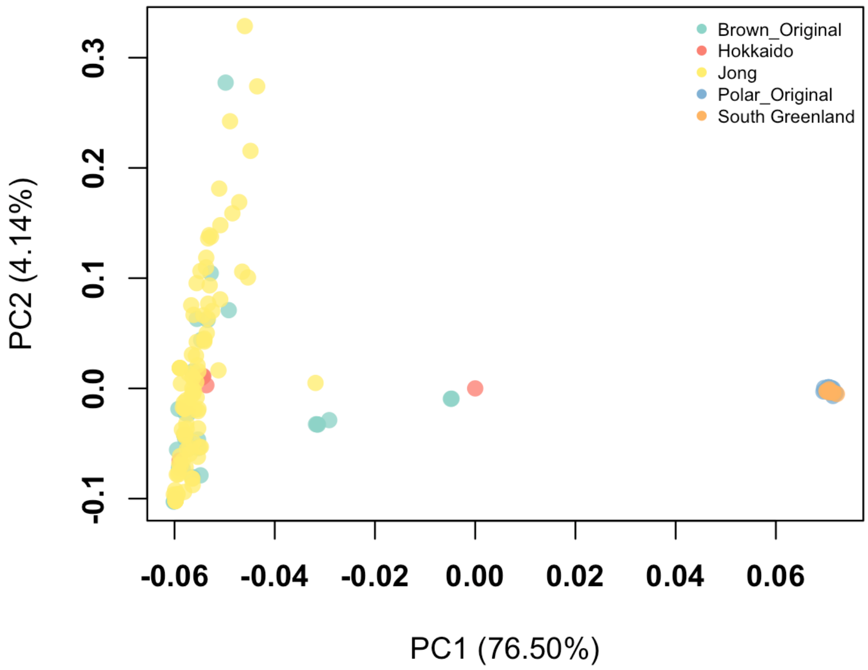

Figure S5. Principal component analysis of gene *COL5A3* and the 50kb flanking regions using all present-day individuals (119 polar bears, 135 brown bears) included in this study. ‘Polar_Original’ and ‘Brown_Original’ refer to the samples from Castruita, Westbury, and Lorenzen 2020. ‘South Greenland’, ‘Hokkaido’, represent new polar bear datasets from Laidre et al 2023 and Endo et al 2021, respectively. ‘Jong’ shows the Holarctic brown bear dataset from de Jong et al 2023.

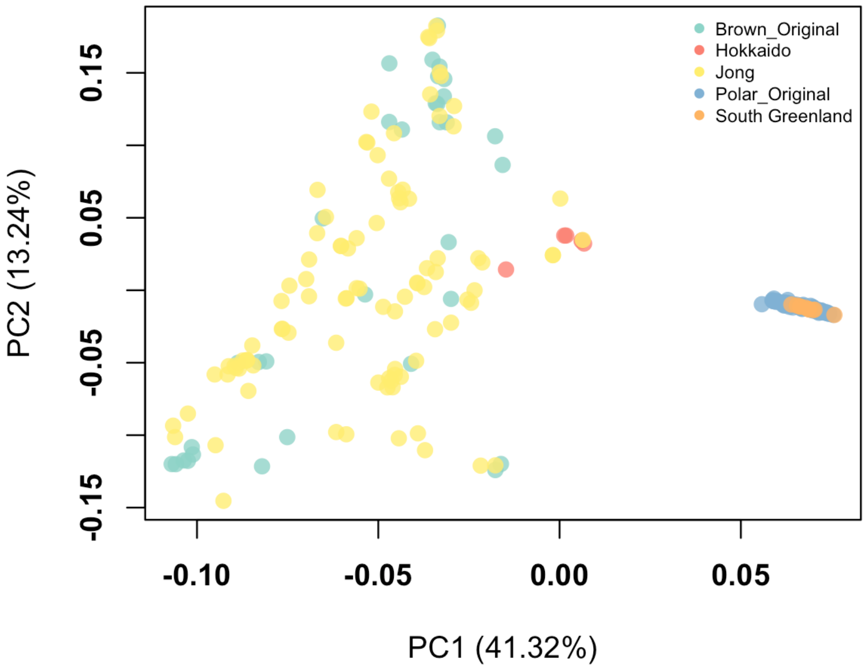

Figure S6. Principal component analysis of gene *CUL7* and the 50kb flanking regions using all present-day individuals (119 polar bears, 135 brown bears) included in this study. ‘Polar_Original’ and ‘Brown_Original’ refer to the samples from Castruita, Westbury, and Lorenzen 2020. ‘South Greenland’, ‘Hokkaido’, represent new polar bear datasets from Laidre et al 2023 and Endo et al 2021, respectively. ‘Jong’ shows the Holarctic brown bear dataset from de Jong et al 2023.

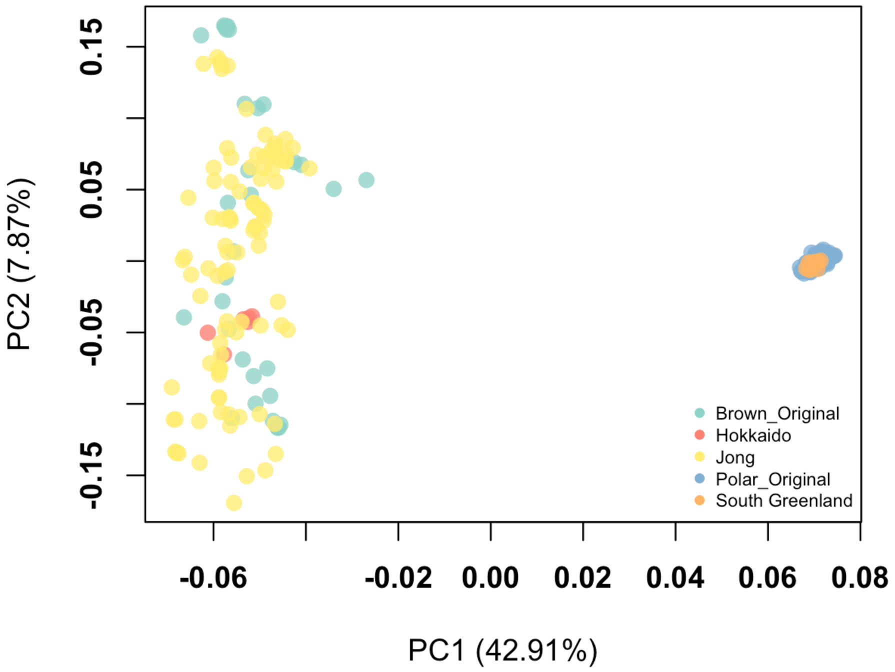

Figure S7. Principal component analysis of gene *FCGBP* and the 50kb flanking regions using all present-day individuals (119 polar bears, 135 brown bears) included in this study. ‘Polar_Original’ and ‘Brown_Original’ refer to the samples from Castruita, Westbury, and Lorenzen 2020. ‘South Greenland’, ‘Hokkaido’, represent new polar bear datasets from Laidre et al 2023 and Endo et al 2021, respectively. ‘Jong’ shows the Holarctic brown bear dataset from de Jong et al 2023.

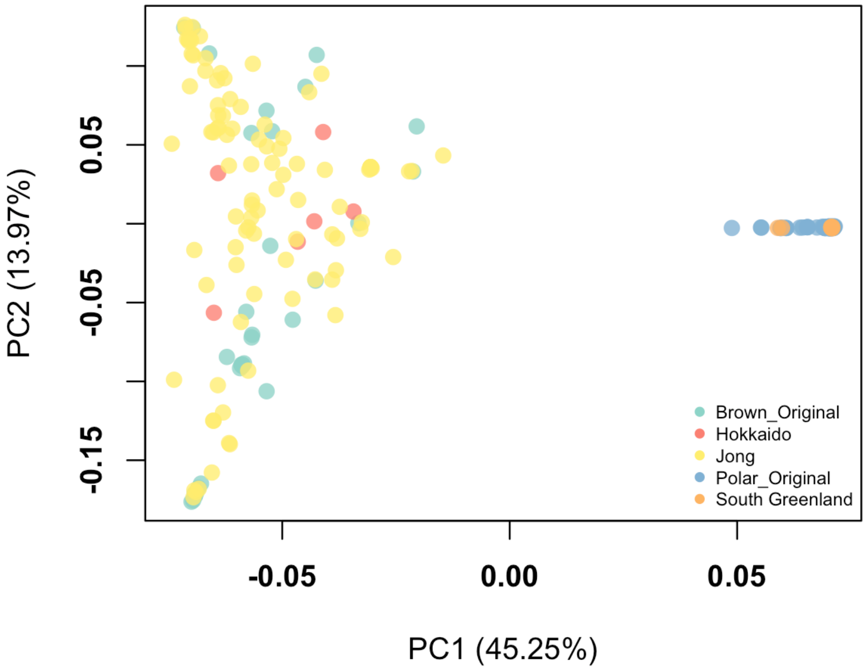

Figure S8. Principal component analysis of gene *LYST* and the 50kb flanking regions using all present-day individuals (119 polar bears, 135 brown bears) included in this study. ‘Polar_Original’ and ‘Brown_Original’ refer to the samples from Castruita, Westbury, and Lorenzen 2020. ‘South Greenland’, ‘Hokkaido’, represent new polar bear datasets from Laidre et al 2023 and Endo et al 2021, respectively. ‘Jong’ shows the Holarctic brown bear dataset from de Jong et al 2023.

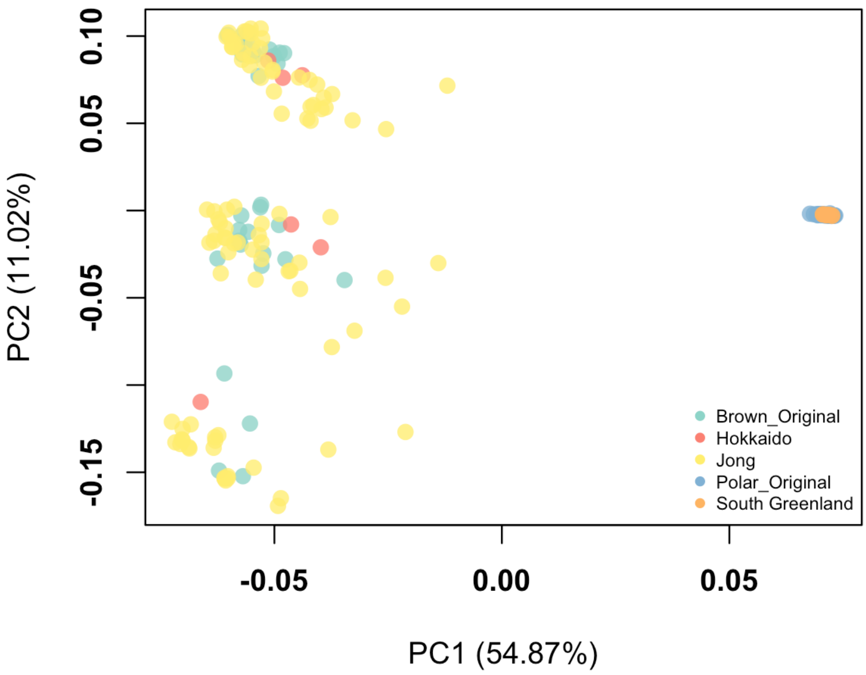

Figure S9. Principal component analysis of gene *LAMC3* and the 50kb flanking regions using all present-day individuals (119 polar bears, 135 brown bears) included in this study. ‘Polar_Original’ and ‘Brown_Original’ refer to the samples from Castruita, Westbury, and Lorenzen 2020. ‘South Greenland’, ‘Hokkaido’, represent new polar bear datasets from Laidre et al 2023 and Endo et al 2021, respectively. ‘Jong’ shows the Holarctic brown bear dataset from de Jong et al 2023.

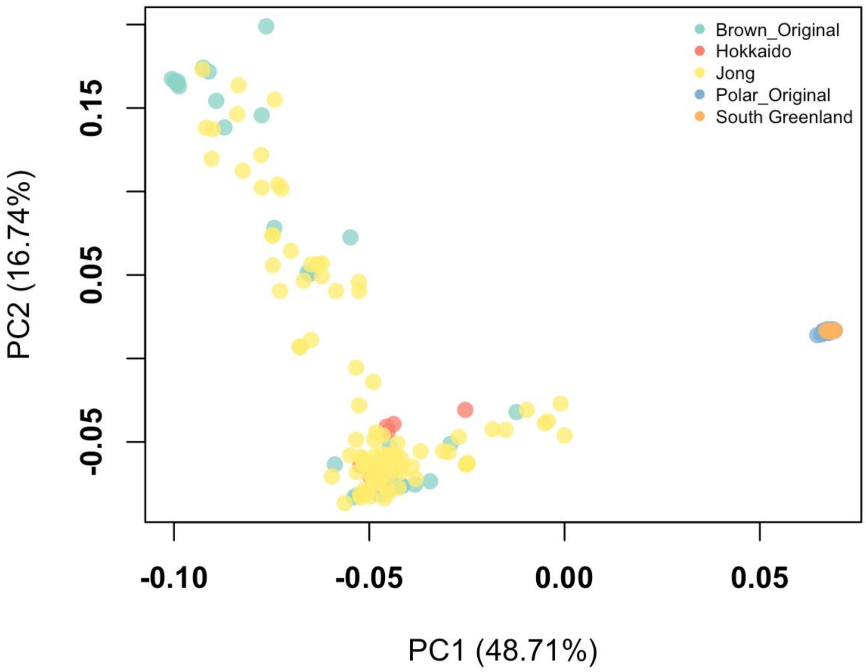

Figure S10. Principal component analysis of gene *POLR1A* and the 50kb flanking regions using all present-day individuals (119 polar bears, 135 brown bears) included in this study. ‘Polar_Original’ and ‘Brown_Original’ refer to the samples from Castruita, Westbury, and Lorenzen 2020. ‘South Greenland’, ‘Hokkaido’, represent new polar bear datasets from Laidre et al 2023 and Endo et al 2021, respectively. ‘Jong’ shows the Holarctic brown bear dataset from de Jong et al 2023.

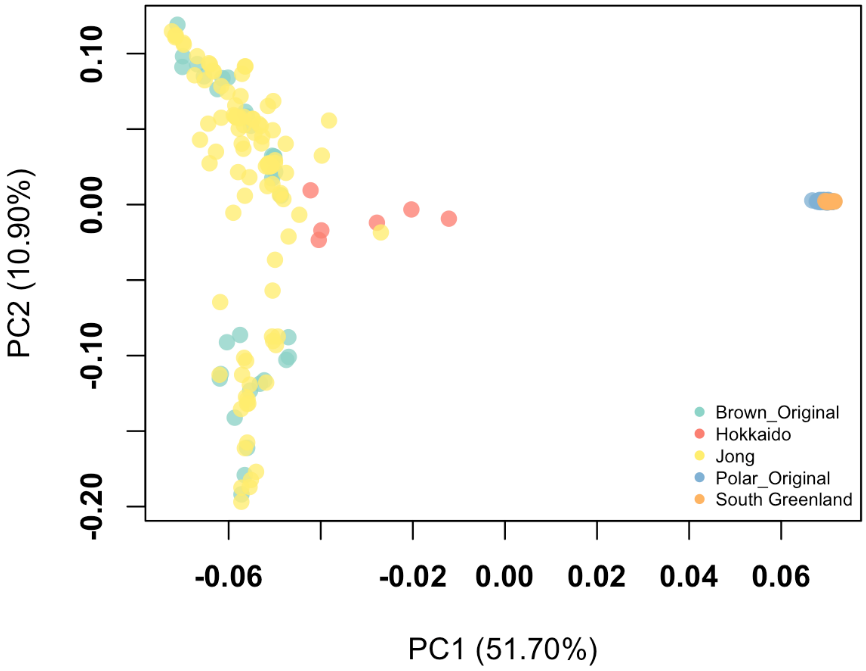

Figure S11. Principal component analysis of gene *TTN* and the 50kb flanking regions using all present-day individuals (119 polar bears, 135 brown bears) included in this study. ‘Polar_Original’ and ‘Brown_Original’ refer to the samples from Castruita, Westbury, and Lorenzen 2020. ‘South Greenland’, ‘Hokkaido’, represent new polar bear datasets from Laidre et al 2023 and Endo et al 2021, respectively. ‘Jong’ shows the Holarctic brown bear dataset from de Jong et al 2023.

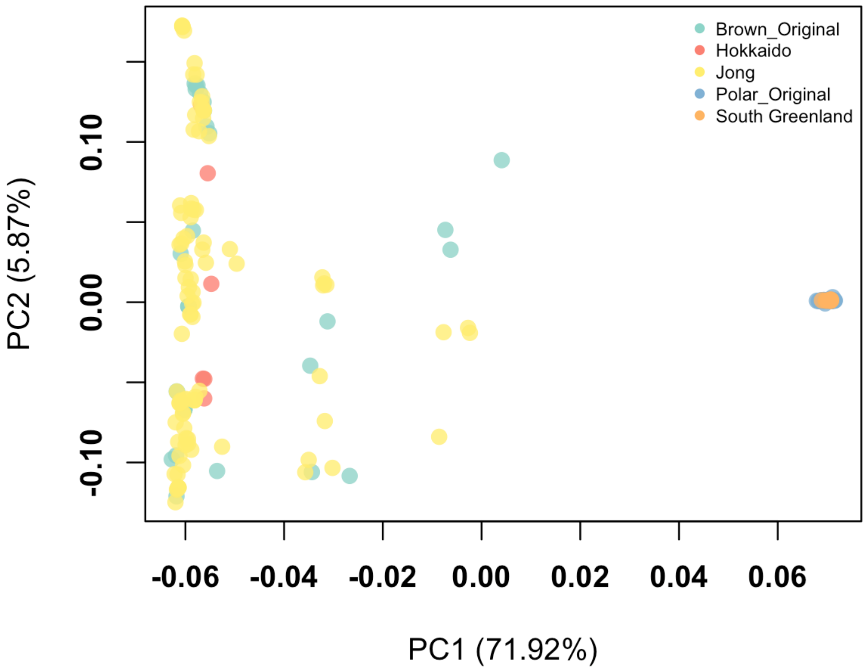

Figure S12. Principal component analysis of gene *XIPR1* and the 50kb flanking regions using all present-day individuals (119 polar bears, 135 brown bears) included in this study. ‘Polar_Original’ and ‘Brown_Original’ refer to the samples from Castruita, Westbury, and Lorenzen 2020. ‘South Greenland’, ‘Hokkaido’, represent new polar bear datasets from Laidre et al 2023 and Endo et al 2021, respectively. ‘Jong’ shows the Holarctic brown bear dataset from de Jong et al 2023.
